# Compliance Modeling and Kinetostatic Analysis of a Generalized 3-PSS Compliant Parallel Micro-Motion Platform

**DOI:** 10.3390/mi15030354

**Published:** 2024-02-29

**Authors:** Jun Ren, Aojie Lan

**Affiliations:** Hubei Key Laboratory of Modern Manufacturing Quantity Engineering, School of Mechanical Engineering, Hubei University of Technology, Wuhan 430068, China; 102110167@hbut.edu.cn

**Keywords:** generalized 3-PSS, coordinate transform method, compliance matrix, kinetostatic performance analysis

## Abstract

In order to expand the range of motion performance of the 3-PSS-compliant parallel micro-motion platform, a variable inclination angle of the mechanism’s guide rails was introduced to construct a category of generalized 3-PSS compliant parallel micro-motion platforms with distinct configurations (exhibiting different motion performances) but identical motion patterns (three translational degrees of freedom). The compliance and kinetostatics of such micro-motion platform are modeled and analyzed. Firstly, the compliance model is established based on the coordinate transformation method. Then, simplifying the micro-motion platform into a spring system, the kinetostatic model in terms of input force–output displacement is established based on the compliance model using the compliance matrix method. For practical application considerations, the kinetostatic model in terms of input displacement–output displacement is further derived based on the input force–output displacement model. Then, the correctness of the established compliance model and kinetostatic model is successively verified through finite element simulation. Finally, using two specified motion trajectories (spatial spiral trajectory and planar circular trajectory) as examples, an analysis is conducted on the influence of guide rail inclination angle variations on the kinetostatic performance of the micro-motion platform. This analysis serves as guidance for the rational design of such micro-motion platforms.

## 1. Introduction

The rapid development of micro-operation or micro-positioning fields has led to increasingly high requirements for mechanism accuracy. Traditional rigid and series mechanisms are no longer able or find it difficult to meet their accuracy requirements [1,2]. On the other hand, compliant parallel mechanisms take into account the advantages of the friction-free, gap-free, assembly-free, and high-precision nature of the compliant mechanism, as well as the advantages of the high stiffness and high load of the parallel mechanism [3,4,5,6]. Compliant parallel mechanisms have been widely used in precision fields such as aerospace, pointing mechanisms, precision manufacturing, fiber optic docking, biomedicine, and other fields [7,8,9,10,11]. Among the emerging compliant parallel mechanisms, low-degree-of-freedom (especially three-degree-of-freedom) mechanisms are favored by many scholars due to their advantages of fewer driving components, more compact structure, and relatively low cost.

Ammar Al-Jodah et al. [12] devised a three-degree-of-freedom XYθ-type large-scale micro-positioning platform with large workspace and high motion accuracy and conducted kinetostatic and kinematic analyses on it. In the context of precision operational environments, Jinhai Gao et al. [13] introduced a low-coupling three-degree-of-freedom-compliant hybrid micromechanical arm for precision operating environments, with a motion range of up to 100 μm in each dimension. Guilian Wang et al. [14] designed a three-degree-of-freedom micro-positioning platform with high positioning accuracy, strong load-bearing capacity, and large motion range, and analyzed its kinematic, kinetostatic, and frequency characteristics. Ning Chen et al. [15] devised a three-degree-of-freedom-compliant nano-displacement mechanism and established its kinetostatic model using the compliance matrix method. Ren and Cao [16] proposed a 3-PSS compliant parallel micro-motion platform with three spatial translational degrees of freedom and conducted dynamic modeling and frequency characteristic analysis on it. However, the three-degree-of-freedom-compliant parallel mechanisms designed in the aforementioned literature often encounter limitations in their motion performance when confronted with more complex working conditions due to their predetermined structural configurations.

The analysis of kinetostatics plays an indispensable role in the study of compliant parallel mechanisms, and it serves as the foundational basis for subsequent dynamic analyses. Due to the coupling between the motion process and the elastic mechanics behavior of the compliant hinges, the kinetostatics of compliant parallel mechanisms cannot be analyzed solely through kinematics or statics, which poses certain challenges. In recent years, scholars have presented various methods for the kinetostatic modeling of compliant mechanisms [17], including the pseudo-rigid body model method [18], Castigliano’s theorems [19], and the compliance matrix method [20]. VenKiteswaran et al. [21] conducted a study on the pseudo-rigid body model of a compliant beam with three rotations and derived its kinematic and static equations based on this model. Guimin Chen et al. [22], combining Castigliano’s first theorem with the Crotti–Engesser theorem, established an energy-based kinetostatic modeling framework suitable for compliant mechanisms. Ren J and Wu [23] proposed a three-degree-of-freedom 3-PSS/S flexible parallel micro-turntable and modeled its kinetostatics using both the pseudo-rigid body model approach and the compliance matrix method. They also compared and analyzed the differences in accuracy between the two kinematic models. Ren J and Li [24] proposed a class of n-4R-compliant parallel pointing mechanisms. They modeled and analyzed the compliance and kinetostatics of this mechanism based on the compliance matrix method. Arredondo-Soto et al. [25] introduced a unified systematic approach based on the compliance matrix method for the kinetostatic modeling of compliant parallel mechanisms. From these references, it can be seen that the compliance matrix method is widely employed in the kinetostatic modeling of compliant parallel mechanisms due to its features such as low computational complexity and the ability to fully consider the deformation of flexible elements in various directions.

This paper takes the 3-PSS compliant parallel mechanism proposed in reference [16] as the prototype and constructs a class of different configurations of the 3-PSS compliant parallel micro-motion platforms with the same motion mode (three translational degrees of freedom) but different motion performances (workspace, motion precision, etc.) by adjusting the guide rail inclination angle of the mechanism. We refer to it as the generalized 3-PSS compliant parallel micro-motion platform in the subsequent text and analyze the compliance and kinetostatics of this generalized micro-motion platform. Due to the established structure of the original 3-PSS compliant parallel micro-motion platform, its motion range and the required input stroke are fixed, and it will be limited to a certain extent in the face of a more complex working environment. At this time, the appropriate guide rail inclination angle can be selected according to the analysis results to improve some specific performances of the original 3-PSS compliant parallel micro-motion platform (such as workspace, motion precision, etc.) to meet specific performance requirements. The rest of the paper is organized as follows: In Section 2, the structural composition of the generalized 3-PSS compliant parallel micro-motion platform is introduced. In Section 3, the compliance of this generalized 3-PSS compliant parallel micro-motion platform is modeled, and the kinetostatic model is subsequently established based on it. The mapping relationship between input displacement and output displacement is further derived based on the relationship between input force and output displacement in the kinetostatic model. In Section 4, the correctness of the compliance model and kinetostatic model is validated through finite element analysis with a set of given parameters. Subsequently, the influence of changes in guide rail inclination angle on the kinetostatic performance of the micro-motion platform is analyzed, providing reference for the design of this type of 3-PSS compliant parallel micro-motion platform to meet specific performance requirements. The conclusions are summarized in Section 5.

## 2. Structure of Micro-Motion Platform

**Figure 1:**
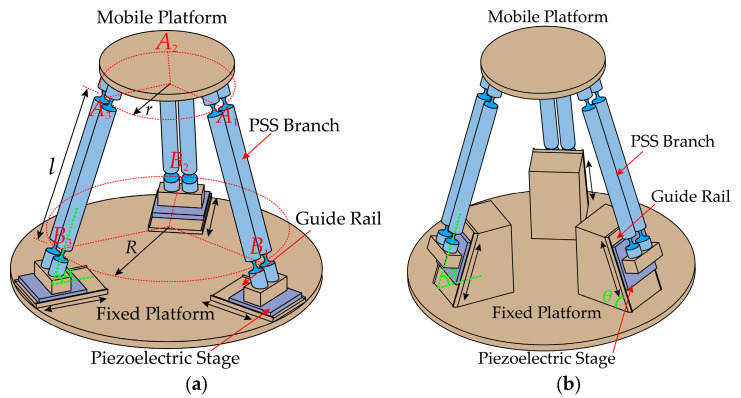
Configurations of the generalized 3-PSS compliant parallel micro-motion platform for different guide rail inclination angles: (**a**) *θ* = 0°; (**b**) *θ* = *α*.

The generalized 3-PSS compliant parallel micro-motion platform consists of a mobile platform, a fixed platform, guide rails, piezoelectric stages, and three PSS (P for the moving slider, driven by the piezoelectric stage; S for compliant spherical hinges) branch chains connecting the mobile and fixed platforms. These three branch chains are circularly evenly distributed, and each branch chain has two identical parallel links, and the two ends are connected to the slider and the mobile platform, respectively, through two uniformly sized compliant spherical hinges. According to the Kutzbach–Grübler formula and the screw theory, it is known that the micro-motion platform possesses three translational degrees of freedom. As shown in Figure 1, points $A_{i}$ and $B_{i}$ (*i* = 1, 2, 3) represent the midpoint of the center line of the compliant spherical hinge at both ends of the links, respectively. The radius of the circle formed by point $A_{i}$ (*i* = 1, 2, 3) is denoted as *r,* while the radius of the circle formed by point $B_{i}$ (*i* = 1, 2, 3) is denoted as *R*. The distance between $A_{i}$ and $B_{i}$ is denoted as *l*. Then, the initial angle between the link and the fixed platform can be determined, as denoted as *α*. The angle between the axis of the guide rail and the fixed platform is denoted as *θ* (referred to as the guide rail inclination angle throughout the text). Assuming that the guide rail is always located below the link, it follows that 0° ≤ *θ* ≤ *α*. Figure 1a,b illustrates the configurations of the mechanism corresponding to the critical cases of *θ* = 0° and *θ* = *α*.

## 3. The Compliance and Kinetostatic Model of the Micro-Motion Platform

### 3.1. Compliance Modeling

Compliance serves as a crucial performance indicator that reflects the ability of a mechanism to resist external loads, and the compliance modeling is the basis for analyzing the kinetostatics. When conducting compliance analysis on the micro-motion platform, it is assumed that all three sliders are fixed. Therefore, while keeping other parameters unchanged, variations in the inclination angle of the guide rail do not affect the compliance of the micro-motion platform. The primary factors affecting the compliance are the structural parameters of the compliant spherical hinges and the dimensional parameters of the micro-motion platform.

**Figure 2:**
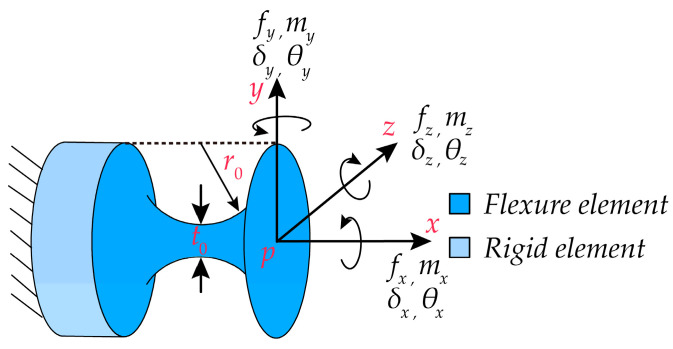
Structure of the right-circular compliant spherical hinge and coordinate system establishment.

As shown in Figure 2, the right-circular compliant spherical hinge with a minimum thickness of $t_{0}$ and a cutting radius of $r_{0}$ is applied to the micro-motion platform. The coordinate system p−xyz is established at the free end face of the compliant spherical hinge. It is assumed that the load vector acting on the free end face of the right-circular compliant spherical hinge is $F={\left[m_{x},m_{y},m_{z},f_{x},f_{y},f_{z}\right]}^{T}$ and the resulting displacement vector is $\Delta ={\left[\theta_{x},\theta_{y},\theta_{z},\delta_{x},\delta_{y},\delta_{z}\right]}^{T}$. Based on linear elasticity and small deformation assumptions, neglecting the small interference generated by deformations in each direction, the displacement–force equation can be obtained according to reference [26] as follows:

(1) $$\Delta =\left[\begin{array}{c} \theta_{x}\\ \theta_{y}\\ \theta_{z}\\ \delta_{x}\\ \delta_{y}\\ \delta_{z} \end{array}\right]=\left[\begin{array}{cccccc} C_{\theta_{x},m_{x}} & 0 & 0 & 0 & 0 & 0\\ 0 & C_{\theta_{y},m_{y}} & 0 & 0 & 0 & C_{\theta_{y},f_{z}}\\ 0 & 0 & C_{\theta_{z},m_{z}} & 0 & C_{\theta_{z},f_{y}} & 0\\ 0 & 0 & 0 & C_{\delta_{x},f_{x}} & 0 & 0\\ 0 & 0 & C_{\delta_{y},m_{z}} & 0 & C_{\delta_{y},f_{y}} & 0\\ 0 & C_{\delta_{z},m_{y}} & 0 & 0 & 0 & C_{\delta_{z},f_{z}} \end{array}\right]\left[\begin{array}{c} m_{x}\\ m_{y}\\ m_{z}\\ f_{x}\\ f_{y}\\ f_{z} \end{array}\right]=CF$$

where ***C*** represents the compliance matrix of the right-circular compliant spherical hinge. It is important to note that the composition of ***C*** depends on the arrangement of the load vector ***F*** and the displacement vector Δ. The calculation of ***C*** can be found in Appendix A.

When calculating compliance in various coordinate systems, it is essential to transform the compliance matrix of the compliant element into the corresponding reference coordinate system through coordinate transformations. From Equation (1), it is known that the compliance matrix of the compliant spherical hinge in its local coordinate system p−xyz is ***C***. Let $C_{p}^{p\prime }$ be the compliance matrix of the compliant spherical hinge in another reference coordinate system $p^{\prime }-x^{\prime }y^{\prime }z^{\prime }$. The relationship for the compliance transformation from its local coordinate system $\left\{p\right\}$ to the reference coordinate system $\left\{p^{\prime }\right\}$ is given by the following:

(2) $$C_{p}^{p^{\prime }}={\left[T\right]}_{p}^{p^{\prime }}C{\left({\left[T\right]}_{p}^{p^{\prime }}\right)}^{T}$$

where ${\left[T\right]}_{p}^{p^{\prime }}$ is a 6 × 6 coordinate transformation matrix, which is represented as follows:

(3) $${\left[T\right]}_{p}^{p^{\prime }}=\left[\begin{array}{cc} {\left[R\right]}_{p}^{p^{\prime }} & 0_{3\times 3}\\ {\left[D\right]}_{p}^{p^{\prime }}{\left[R\right]}_{p}^{p^{\prime }} & {\left[R\right]}_{p}^{p^{\prime }} \end{array}\right]$$

where ${\left[R\right]}_{p}^{p^{\prime }}$ represents the rotation matrix of the local coordinate system $\left\{p\right\}$ with respect to the reference coordinate system $\left\{p^{\prime }\right\}$, and ${\left[D\right]}_{p}^{p^{\prime }}$ is the antisymmetric matrix of the position vector $d_{p}^{p^{\prime }}={\left[\begin{array}{ccc} x & y & z \end{array}\right]}^{T}$ in the local coordinate system $\left\{p\right\}$ relative to the reference coordinate system $\left\{p^{\prime }\right\}$, defined as follows:

(4) $${\left[D\right]}_{p}^{p^{\prime }}=\left[\begin{array}{ccc} 0 & -z & y\\ z & 0 & -x\\ -y & x & 0 \end{array}\right]$$

**Figure 3:**
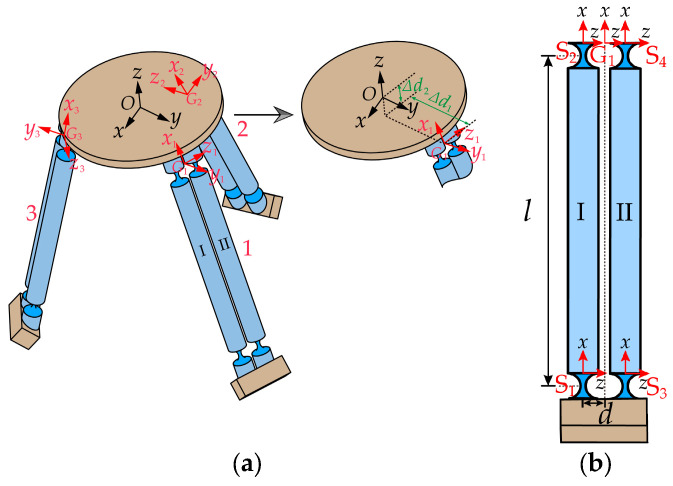
(**a**) Establishment of the global coordinate system for the micro-motion platform and local coordinate systems for each branch chain. (**b**) Local coordinate system for branch chain 1.

The establishment of coordinate systems for compliance analysis in the micro-motion platform is illustrated in Figure 3a. The global coordinate system O−xyz (denoted as $\left\{O\right\}$) is established at the center of upper surface of the mobile platform, and the local coordinate systems $G_{i}-xyz$ for each branch chain are established at the midpoint of the line connecting the centers of the end faces of two compliant spherical hinges connected to the mobile platform. First, the compliance of a single branch chain of the micro-motion platform needs to be calculated. As depicted in Figure 3b, PSS branch chain 1 is formed by the parallel connection of two identical PSS links, I and II. Local coordinate systems $S_{1}-xyz$, $S_{2}-xyz$, $S_{3}-xyz$, and $S_{4}-xyz$ (hereinafter referred to as $\left\{S_{1}\right\}$, $\left\{S_{2}\right\}$, $\left\{S_{3}\right\}$, $\left\{S_{4}\right\}$) are individually established at the centers of end surfaces of the four compliant spherical hinge surfaces. The orientations of these four coordinate systems are aligned with the directions of the $G_{1}-xyz$ coordinate system. The horizontal distance between the origins of the local coordinate system $S_{i}-xyz$ and the local coordinate system $G_{1}-xyz$ is denoted as *d*.

According to Equation (2), the compliance matrices of the compliant spherical hinges $S_{1}$ and $S_{3}$ in the local coordinate systems $\left\{S_{2}\right\}$ and $\left\{S_{4}\right\}$, respectively, are denoted as $C_{S_{1}}^{S_{2}}$ and $C_{S_{3}}^{S_{4}}$. The computational outcomes are expressed as expressed in Equation (5).

(5) $$\left\{\begin{array}{l} C_{S_{1}}^{S_{2}}={\left[T\right]}_{S_{1}}^{S_{2}}C{\left({\left[T\right]}_{S_{1}}^{S_{2}}\right)}^{T}\\ C_{S_{3}}^{S_{4}}={\left[T\right]}_{S_{3}}^{S_{4}}C{\left({\left[T\right]}_{S_{3}}^{S_{4}}\right)}^{T} \end{array}\right.$$

where matrix ***C*** is given in Equation (1). ${\left[T\right]}_{S_{1}}^{S_{2}}$ and ${\left[T\right]}_{S_{3}}^{S_{4}}$ can be computed using Equations (3) and (4), with the results as follows:

$$\left\{\begin{array}{c} {\left[T\right]}_{S_{1}}^{S_{2}}=\left[\begin{array}{cc} {\left[R\right]}_{S_{1}}^{S_{2}} & 0_{3\times 3}\\ {\left[D\right]}_{S_{1}}^{S_{2}}{\left[R\right]}_{S_{1}}^{S_{2}} & {\left[R\right]}_{S_{1}}^{S_{2}} \end{array}\right],{\left[R\right]}_{S_{1}}^{S_{2}}=I_{3\times 3},{\left[D\right]}_{S_{1}}^{S_{2}}=\left[\begin{array}{ccc} 0 & 0 & 0\\ 0 & 0 & l\\ 0 & -l & 0 \end{array}\right]\\ {\left[T\right]}_{S_{3}}^{S_{4}}=\left[\begin{array}{cc} {\left[R\right]}_{S_{3}}^{S_{4}} & 0_{3\times 3}\\ {\left[D\right]}_{S_{3}}^{S_{4}}{\left[R\right]}_{S_{3}}^{S_{4}} & {\left[R\right]}_{S_{3}}^{S_{4}} \end{array}\right],{\left[R\right]}_{S_{3}}^{S_{4}}=I_{3\times 3},{\left[D\right]}_{S_{3}}^{S_{4}}=\left[\begin{array}{ccc} 0 & 0 & 0\\ 0 & 0 & l\\ 0 & -l & 0 \end{array}\right] \end{array}\right.$$

Applying the principle of compliance superposition for series-connected compliant modules [25], the overall compliance matrices of PSS links I and II in the local coordinate systems $\left\{S_{2}\right\}$ and $\left\{S_{4}\right\}$, respectively, are represented as $C^{S_{2}}$ and $C^{S_{4}}$.

(6) $$\left\{\begin{array}{l} C^{S_{2}}=C_{S_{1}}^{S_{2}}+C\\ C^{S_{4}}=C_{S_{3}}^{S_{4}}+C \end{array}\right.$$

Similarly, the compliance matrices of PSS links I and II in the local coordinate systems $\left\{S_{2}\right\}$ and $\left\{S_{4}\right\}$ can be transformed to the local coordinate system $\left\{G_{1}\right\}$ through coordinate transformations. Applying the stiffness superposition principle for parallel-connected compliant modules [25], the overall compliance matrix of PSS branch chain 1 in the local coordinate system $\left\{G_{1}\right\}$ is represented as $C^{G_{1}}$.

(7) $$C^{G_{1}}={\left({\left({\left[T\right]}_{S_{2}}^{G_{1}}C^{S_{2}}{\left({\left[T\right]}_{S_{2}}^{G_{1}}\right)}^{T}\right)}^{-1}+{\left({\left[T\right]}_{S_{4}}^{G_{1}}C^{S_{4}}{\left({\left[T\right]}_{S_{4}}^{G_{1}}\right)}^{T}\right)}^{-1}\right)}^{-1}$$

where

T=S2G1RS2G103×3DS2G1RS2G1RS2G1,R=S2G1I3×3,D=S2G10d0−d00000T=S4G1RS4G103×3DS4G1RS4G1RS4G1,R=S4G1I3×3,D=S4G10−d0d00000

By further coordinate transformation, the compliance matrix of PSS branch chain 1 in the global coordinate system $\left\{O\right\}$ can be obtained as $C_{pss1}$.

(8) $$C_{pss1}={\left[T\right]}_{G_{1}}^{O}C^{G_{1}}{\left({\left[T\right]}_{G_{1}}^{O}\right)}^{T}$$

where

$${\left[T\right]}_{G_{1}}^{O}=\left[\begin{array}{cc} {\left[R\right]}_{G_{1}}^{O} & 0_{3\times 3}\\ {\left[D\right]}_{G_{1}}^{O}{\left[R\right]}_{G_{1}}^{O} & {\left[R\right]}_{G_{1}}^{O} \end{array}\right],{\left[R\right]}_{G_{1}}^{O}=\left[R_{x,\left(\left(\pi /2\right)-\alpha \right)}\right]\left[R_{y,-\pi /2}\right],{\left[D\right]}_{G_{1}}^{O}=\left[\begin{array}{ccc} 0 & -\Delta d_{2} & \Delta d_{1}\\ \Delta d_{2} & 0 & 0\\ -\Delta d_{1} & 0 & 0 \end{array}\right]$$

$\Delta d_{1}$ and $\Delta d_{2}$, respectively, represent the position vectors of the local coordinate system $\left\{\left.G_{1}\right\}\right.$ relative to the global coordinate system $\left\{O\right\}$ in the *y* and *z* directions, as shown in Figure 3a.

Due to the fact that the three PSS branch chains of the micro-motion platform have identical structures and are evenly distributed in the circumferential direction, it is sufficient to rotate the compliance matrix of PSS branch chain 1 about the *z*-axis of the global coordinate system $\left\{O\right\}$ by 120° and 240° to obtain the compliance matrices $C_{pss2}$ and $C_{pss3}$ for PSS branch chains 2 and 3 in the global coordinate system $\left\{O\right\}$.

(9) $$\left\{\begin{array}{c} C_{pss2}=\left[T_{R,2\pi /3}\right]C_{pss1}{\left(\left[T_{R,2\pi /3}\right]\right)}^{T}\\ C_{pss3}=\left[T_{R,4\pi /3}\right]C_{pss1}{\left(\left[T_{R,4\pi /3}\right]\right)}^{T} \end{array}\right.$$

where

$$\begin{array}{c} \left[T_{R,2\pi /3}\right]=\left[\begin{array}{cc} R_{z,2\pi /3} & 0_{3\times 3}\\ 0_{3\times 3} & R_{z,2\pi /3} \end{array}\right]\\ \left[T_{R,4\pi /3}\right]=\left[\begin{array}{cc} R_{z,4\pi /3} & 0_{3\times 3}\\ 0_{3\times 3} & R_{z,4\pi /3} \end{array}\right] \end{array}$$

Applying the stiffness superposition principle for parallel-connected compliant modules, the overall compliance matrix of the generalized 3-PSS compliant parallel micro-motion platform can be then obtained as follows:

(10) $$C_{total}={\left({\left(C_{pss1}\right)}^{-1}+{\left(C_{pss2}\right)}^{-1}+{\left(C_{pss3}\right)}^{-1}\right)}^{-1}$$

### 3.2. Kinetostatic Modeling

**Figure 4:**
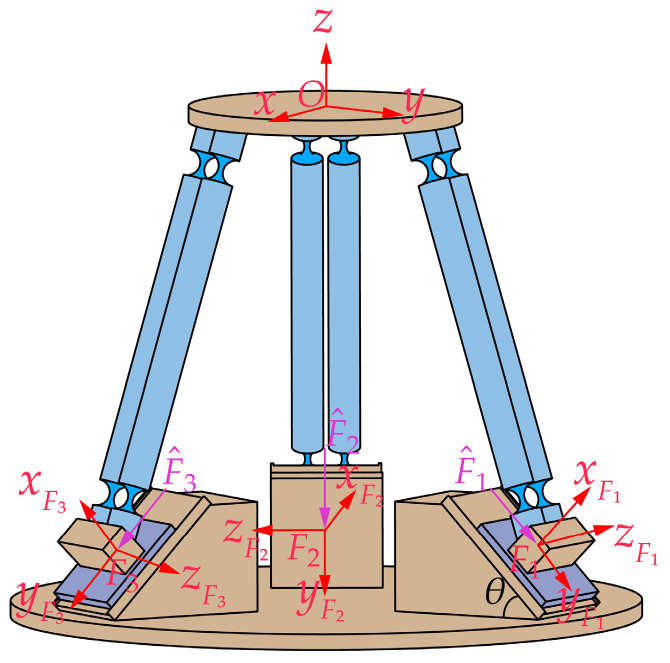
Kinetostatic model of the generalized 3-PSS micro-motion platform.

Based on the compliance of each PSS branch chain in the global coordinate system $\left\{O\right\}$ established in Section 3.1, the kinetostatic model of the generalized 3-PSS compliant parallel micro-motion platform is further investigated through the compliance matrix method. As depicted in Figure 4, we establish force coordinate systems $F_{i}-x_{F_{i}}y_{F_{i}}z_{F_{i}}$ (*i* = 1, 2, 3) at the center of the bottom surfaces of three sliders. We designate the input forces as ${\hat{F}}_{i}={\left[m_{x},m_{y},m_{z},f_{x},f_{y},f_{z}\right]}^{T}$ (*i* = 1, 2, 3) and denote the resulting displacement of the mobile platform in the global coordinate system $\left\{O\right\}$ as ${\hat{U}}_{O}={\left[\theta_{x},\theta_{y},\theta_{z},\delta_{x},\delta_{y},\delta_{z}\right]}^{T}$. Assuming deformation within the linear range, the output displacement ${\hat{U}}_{O}$ of the mobile platform can be obtained by superimposing the displacements ${\hat{U}}_{i}$ (*i* = 1, 2, 3) individually generated by the three input forces ${\hat{F}}_{i}$ (*i* = 1, 2, 3).

**Figure 5:**
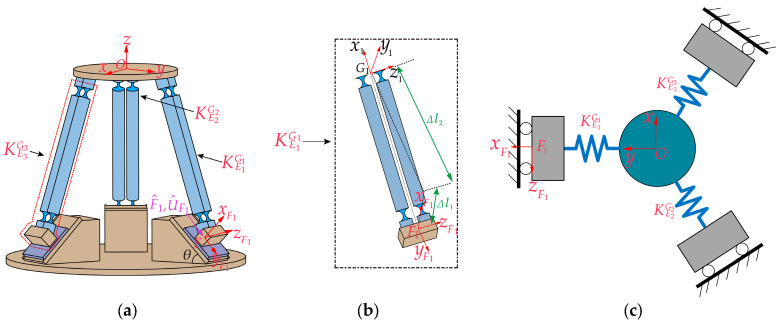
(**a**) Only ${\hat{F}}_{1}$ acting on the micro-motion platform; (**b**) equivalent stiffness of PSS branch chain 1; (**c**) simplified spring system.

We assume that the micro-motion platform is only subjected to the action of ${\hat{F}}_{1}$, as shown in Figure 5a. Introducing the concept of equivalent stiffness, we establish the same local coordinate systems $G_{1}-x_{1}y_{1}z_{1}$ as in Section 3.1, as depicted in Figure 5b. For the convenience of analysis, the micro-motion platform is simplified as a spring system, as shown in Figure 5c. The equivalent stiffness matrices of the three PSS branch chains in the local coordinate system $G_{i}-x_{i}y_{i}z_{i}$ are denoted as $K_{E_{1}}^{G_{1}}$, $K_{E_{2}}^{G_{2}}$, and $K_{E_{3}}^{G_{3}}$, respectively.

According to Hooke’s law, the governing equation for the elastic deformation of the spring system can be expressed as follows:

(11) $$\left[\begin{array}{cc} {\left(K_{OO}\right)}_{F_{1}} & K_{OF_{1}}\\ K_{F_{1}O} & K_{F_{1}F_{1}} \end{array}\right]\left[\begin{array}{c} {\hat{U}}_{1}\\ {\hat{U}}_{F_{1}} \end{array}\right]=\left[\begin{array}{c} {\hat{F}}_{O}\\ {\hat{F}}_{1} \end{array}\right]$$

where ${\hat{U}}_{1}$ represents the displacement of the center point O of the mobile platform in the coordinate system $\left\{O\right\}$ when force ${\hat{F}}_{1}$ acts alone. ${\hat{U}}_{F_{1}}$ represents the displacement of slider 1 in the coordinate system $\left\{\left.F_{1}\right\}\right.$. ${\hat{F}}_{O}$ represents the force applied at point O. The stiffness matrices ${\left(K_{OO}\right)}_{F_{1}}$, $K_{OF_{1}}$, $K_{F_{1}O}$, and $K_{F_{1}F_{1}}$ are calculated as follows:

(12) $$\left\{\begin{array}{l} {\left(K_{OO}\right)}_{F_{1}}=K_{E_{1}}^{O}+K_{E_{2}}^{O}+K_{E_{3}}^{O}\\ K_{F_{1}F_{1}}=K_{E_{1}}^{F_{1}}\\ K_{OF_{1}}=-K_{E_{1}}^{O,F_{1}}\\ K_{F_{1}O}=-K_{E_{1}}^{F_{1},O} \end{array}\right.$$

where the superscripts $F_{1}$ and ***O*** indicate that the stiffness matrix is relative to the coordinate systems $\left\{\left.F_{1}\right\}\right.$ and $\left\{\left.O\right\}\right.$, respectively. The double superscripts in the stiffness matrix $K_{E_{1}}^{O,F_{1}}$ indicate that the forces $\hat{F}$ and displacements $\hat{U}$ are located in the coordinate systems $\left\{\left.O\right\}\right.$ and $\left\{\left.F_{1}\right\}\right.$, respectively. The same principle applies to the representation of the stiffness matrix $K_{E_{1}}^{F_{1},O}$. $K_{E_{1}}^{O}$, $K_{E_{2}}^{O}$, and $K_{E_{3}}^{O}$ represent the equivalent stiffness of the three PSS branch chains relative to the global coordinate system $\left\{\left.O\right\}\right.$ and can be calculated using Equations (8) and (9) as follows:

(13) $$\left\{\begin{array}{c} K_{E_{1}}^{O}={\left(C_{pss1}\right)}^{-1}\\ K_{E_{2}}^{O}={\left(C_{pss2}\right)}^{-1}\\ K_{E_{3}}^{O}={\left(C_{pss3}\right)}^{-1} \end{array}\right.$$

The stiffness matrices $K_{E1}^{F_{1}}$, $K_{OF_{1}}$, and $K_{F_{1}O}$ can be obtained through matrix transformation using the equivalent stiffness matrix $K_{E_{1}}^{G_{1}}$, and the calculation is as follows:

(14) $$\left\{\begin{array}{l} K_{E_{1}}^{F_{1}}={\left({\left[T\right]}_{G_{1}}^{F_{1}}\right)}^{-T}\left[K_{E_{1}}^{G_{1}}\right]{\left({\left[T\right]}_{G_{1}}^{F_{1}}\right)}^{-1}\\ K_{OF_{1}}=-{\left({\left[T\right]}_{G_{1}}^{O}\right)}^{-T}\left[K_{E_{1}}^{G_{1}}\right]{\left({\left[T\right]}_{G_{1}}^{F_{1}}\right)}^{-1}\\ K_{F_{1}O}=-{\left({\left[T\right]}_{G_{1}}^{F_{1}}\right)}^{-T}\left[K_{E_{1}}^{G_{1}}\right]{\left({\left[T\right]}_{G_{1}}^{O}\right)}^{-1} \end{array}\right.$$

where

$${\left[T\right]}_{G_{1}}^{F_{1}}=\left[\begin{array}{cc} {\left[R\right]}_{G_{1}}^{F_{1}} & 0_{3\times 3}\\ {\left[D\right]}_{G_{1}}^{F_{1}}{\left[R\right]}_{G_{1}}^{F_{1}} & {\left[R\right]}_{G_{1}}^{F_{1}} \end{array}\right]\;,\;{\left[D\right]}_{G_{1}}^{F_{1}}=\left[\begin{array}{ccc} 0 & 0 & \Delta l_{2}\\ 0 & 0 & -\Delta l_{1}\\ -\Delta l_{2} & \Delta l_{1} & 0 \end{array}\right],\;{\left[R\right]}_{G_{1}}^{F_{1}}=\left[R_{z,-\left(\left(\pi /2\right)-\alpha +\theta \right)}\right]\;,$$

$\Delta l_{1}$ and $\Delta l_{2}$, respectively, represent the position vectors of the local coordinate system $\left\{\left.G_{1}\right\}\right.$ relative to the force coordinate system $\left\{\left.F_{1}\right\}\right.$ in the *x* and *y* directions, as shown in Figure 5b. The matrix ${\left[T\right]}_{G_{1}}^{O}$ is given by Equation (8), and the stiffness matrix $K_{E_{1}}^{G_{1}}$ can be obtained from Equation (7).

(15) $$K_{E_{1}}^{G_{1}}={\left(C^{G_{1}}\right)}^{-1}$$

When the mobile platform is not subjected to external forces (${\hat{F}}_{O}=0$), the following can be extracted from Equation (11):

(16) $${\hat{U}}_{1}=-{\left({\left(K_{OO}\right)}_{F_{1}}-K_{OF_{1}}K_{F_{1}F_{1}}^{-1}K_{F_{1}O}\right)}^{-1}\left(K_{OF_{1}}K_{F_{1}F_{1}}^{-1}\right)\cdot {\hat{F}}_{1}$$

Similarly, when the input forces ${\hat{F}}_{i}$ (*i* = 1, 2, 3) act alone, analogous conclusions can be derived.

(17) $${\hat{U}}_{i}=J_{F_{i}}^{O}\cdot {\hat{F}}_{i}\;(i\;=\;1,\;2,\;3)$$

where

(18) $$J_{F_{i}}^{O}=-{\left({\left(K_{OO}\right)}_{F_{i}}-K_{OF_{i}}K_{F_{i}F_{i}}^{-1}K_{F_{i}O}\right)}^{-1}\left(K_{OF_{i}}K_{F_{i}F_{i}}^{-1}\right)\;(i\;=\;1,\;2,\;3)$$

Due to the circularly even distribution of the three PSS branch chains, the stiffness matrices in Equation (18) can be easily obtained by rotating the stiffness matrices in Equation (12) around the global coordinate system $\left\{O\right\}$’s *z*-axis by 120° and 240°. The computational formulas are as follows:

(19) $$\left\{\begin{array}{l} {\left(K_{OO}\right)}_{F_{2}}=\left[T_{R,2\pi /3}\right]\left[{\left(K_{OO}\right)}_{F_{1}}\right]{\left(\left[T_{R,2\pi /3}\right]\right)}^{T}\\ {\left(K_{OO}\right)}_{F_{3}}=\left[T_{R,4\pi /3}\right]\left[{\left(K_{OO}\right)}_{F_{1}}\right]{\left(\left[T_{R,4\pi /3}\right]\right)}^{T}\\ K_{F_{2}F_{2}}=K_{F_{3}F_{3}}=K_{F_{1}F_{1}}\\ K_{OF_{2}}=\left[T_{R,2\pi /3}\right]\left[K_{OF_{1}}\right]\\ K_{OF_{3}}=\left[T_{R,4\pi /3}\right]\left[K_{OF_{1}}\right]\\ K_{F_{2}O}=\left[K_{F_{1}O}\right]{\left(\left[T_{R,2\pi /3}\right]\right)}^{T}\\ K_{F_{3}O}=\left[K_{F_{1}O}\right]{\left(\left[T_{R,4\pi /3}\right]\right)}^{T} \end{array}\right.$$

In accordance with the principle of superposition, the displacement of the mobile platform ${\hat{U}}_{O}$ can be obtained through the summation of ${\hat{U}}_{1}$, ${\hat{U}}_{2}$, and ${\hat{U}}_{3}$.

(20) $${\hat{U}}_{O}=\sum_{i=1}^{3}{\hat{U}}_{i}=\left[\begin{array}{ccc} J_{F_{1}}^{O} & J_{F_{2}}^{O} & J_{F_{3}}^{O} \end{array}\right]\cdot \left[\begin{array}{c} {\hat{F}}_{1}\\ {\hat{F}}_{2}\\ {\hat{F}}_{3} \end{array}\right]$$

Equation (20) establishes the mapping relationship between the input forces and the output displacement of the generalized 3-PSS compliant parallel micro-motion platform. In a practical situation, the three sliders are driven by piezoelectric stages, and the forces acting on the sliders are difficult to determine (especially the five forces or moments in non-motion directions), while the input displacements of the sliders are readily obtainable. Hence, it is necessary to further derive the kinetostatic model in terms of input displacement–output displacement based on the foundation of the input force–output displacement model.

Assuming the mobile platform is free from external forces (${\hat{F}}_{O}=0$), we can extract the relationship between the input force ${\hat{F}}_{1}$ and output displacement ${\hat{U}}_{F_{1}}$ from the governing Equation (11) as follows:

(21) $${\hat{F}}_{1}=\left(K_{F_{1}F_{1}}-K_{F_{1}O}{\left(K_{OO}\right)}_{F_{1}}^{-1}K_{OF_{1}}\right)\cdot {\hat{U}}_{F_{1}}$$

Similarly, when ${\hat{F}}_{i}$ (for *i* = 1, 2, 3) acts independently, one can obtain the following:

(22) $${\hat{F}}_{i}=\left(K_{F_{i}F_{i}}-K_{F_{i}O}{\left(K_{OO}\right)}_{F_{i}}^{-1}K_{OF_{i}}\right)\cdot {\hat{U}}_{F_{i}}\;(i\;=\;1,\;2,\;3)$$

Thus, substituting Equation (22) into Equation (20) yields the kinetostatic model of the micro-motion platform in terms of input displacement–output displacement.

(23) $${\hat{U}}_{O}=\sum_{i=1}^{3}{\hat{U}}_{i}=\left[\begin{array}{ccc} J_{U_{1}}^{O} & J_{U_{2}}^{O} & J_{U_{3}}^{O} \end{array}\right]\cdot \left[\begin{array}{c} {\hat{U}}_{F_{1}}\\ {\hat{U}}_{F_{2}}\\ {\hat{U}}_{F_{3}} \end{array}\right]$$

where

(24) $$J_{U_{i}}^{O}=-{\left({\left(K_{OO}\right)}_{F_{i}}-K_{OF_{i}}K_{F_{i}F_{i}}^{-1}K_{F_{i}O}\right)}^{-1}\left(K_{OF_{i}}K_{F_{i}F_{i}}^{-1}\right)\left(K_{F_{i}F_{i}}-K_{F_{i}O}{\left(K_{OO}\right)}_{F_{i}}^{-1}K_{OF_{i}}\right)\;(i\;=\;1,\;2,\;3)$$

Due to the absence of displacement in the non-motion directions of the sliders driven by the piezoelectric stage, ${\hat{U}}_{F_{i}}={\left[0,0,0,0,\delta_{y_{i}},0\right]}^{T}$. Therefore, Equation (23) can be further simplified to the following:

(25) $$\left[\begin{array}{c} \delta_{X}\\ \delta_{Y}\\ \delta_{Z} \end{array}\right]=\left[\begin{array}{ccc} {\left(J_{U_{1}}^{O}\right)}_{\left[rows4\ldots 6,col5\right]} & {\left(J_{U_{2}}^{O}\right)}_{\left[rows4\ldots 6,col5\right]} & {\left(J_{U_{3}}^{O}\right)}_{\left[rows4\ldots 6,col5\right]} \end{array}\right]\cdot \left[\begin{array}{c} \delta_{y_{1}}\\ \delta_{y_{2}}\\ \delta_{y_{3}} \end{array}\right]$$

where ${\left(J_{U_{i}}^{O}\right)}_{\left[rows4\ldots 6,col5\right]}$ (*i* = 1, 2, 3) represents the last three elements of the fifth column of the mapping matrix $J_{U_{i}}^{O}$ (*i* = 1, 2, 3).

## 4. Finite Element Verification and Analysis

In this section, the compliance model and kinetostatic model of the generalized 3-PSS compliant parallel micro-motion platform established in Section 3 are verified firstly through finite element simulation. Then, a further analysis is conducted on the impact of the variation in guide rail inclination angle on the kinetostatic performance of the micro-motion platform.

### 4.1. Compliance Verification

The provided structural parameters for the generalized 3-PSS compliant parallel micro-motion platform model are presented in Table 1. According to these parameters, the angle *α* between the link and the fixed platform can be computed to be approximately 72°. In accordance with the structural design feature, the guide rail inclination angle *θ* should satisfy 0° ≤ *θ* ≤ *α*. In this simulation case, the guide rail inclination angle is chosen to be 45°. The position vectors $\Delta d_{1}$ and $\Delta d_{2}$ can be directly measured in the 3D model constructed, yielding values of 24.23 mm and −8.12 mm, respectively. The material utilized for the compliant spherical hinge is beryllium bronze (CuBe_2_), and its material properties and dimensional parameters are presented in Table 2.

By substituting the parameters from Table 1 and Table 2 into Equation (10), the overall compliance matrix for the micro-motion platform can be calculated as follows:

$$C_{total}^{AN}=\left[\begin{array}{cccccc} 1.0912\times 10^{-3} & 0 & 0 & 0 & 7.2685\times 10^{-5} & 0\\ 0 & 1.0912\times 10^{-3} & 0 & -7.2685\times 10^{-5} & 0 & 0\\ 0 & 0 & 5.4606\times 10^{-3} & 0 & 0 & 0\\ 0 & -7.2685\times 10^{-5} & 0 & 4.9958\times 10^{-6} & 0 & 0\\ 7.2685\times 10^{-5} & 0 & 0 & 0 & 4.9958\times 10^{-6} & 0\\ 0 & 0 & 0 & 0 & 0 & 8.0793\times 10^{-9} \end{array}\right]$$

The 3 × 3 sub-matrix in the upper-left corner of $C_{total}^{AN}$ represents the rotational compliance matrix, measured in rad/(N·m). The 3 × 3 sub-matrix in the lower-right corner represents the translational compliance matrix, measured in m/N. The 3 × 3 sub-matrices in the lower-left and upper-right corners represent the coupling compliance matrices, measured in m/(N·m) and rad/N, respectively. It is evident that the micro-motion platform exhibits identical angular and linear compliance in the *x* and *y* directions, while it possesses the maximum angular compliance and the minimum linear compliance in the *z* direction.

**Figure 6:**
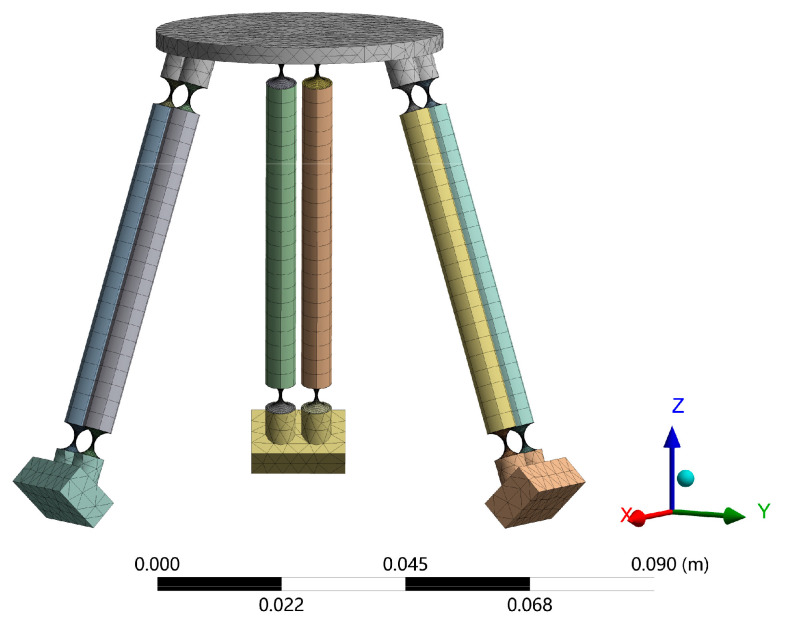
Finite element model of micro-motion platform.

The physical model of the generalized 3-PSS compliant parallel micro-motion platform is constructed based on the parameters in Table 1 and Table 2, and then, the model is imported into ANSYS Workbench 19.2 software for finite element simulation. The mesh partition is depicted in Figure 6. The compliant spherical hinges are meshed using hexahedral structures with the element size of 1 mm and concentrated deformation areas with a size of 0.3 mm. For other components with high stiffness, such as the links and the mobile platform, mesh partition is conducted with an element size of 3 mm. The coordinate system setting in the finite element model remains consistent with that depicted in Figure 3a.

In the finite element simulation, the degrees of freedom for all three input sliders are constrained. Then, unit force or moment is applied at the center of the upper surface of the mobile platform along each coordinate axis direction to obtain the linear or angular compliance of the micro-motion platform in each coordinate axis direction. The simulation results are as follows:

$$C_{total}^{FE}=\left[\begin{array}{cccccc} 1.1747\times 10^{-3} & 0 & 0 & 0 & 7.8248\times 10^{-5} & 0\\ 0 & 1.1747\times 10^{-3} & 0 & -7.8248\times 10^{-5} & 0 & 0\\ 0 & 0 & 5.8785\times 10^{-3} & 0 & 0 & 0\\ 0 & -7.8248\times 10^{-5} & 0 & 5.3785\times 10^{-6} & 0 & 0\\ 7.8248\times 10^{-5} & 0 & 0 & 0 & 5.3785\times 10^{-6} & 0\\ 0 & 0 & 0 & 0 & 0 & 8.7215\times 10^{-9} \end{array}\right]$$

The comparison of the theoretical analysis results (AN) and the finite element simulation results (FE) in the main diagonal elements is presented in Table 3. The results indicate that the theoretical calculations are in good agreement with the simulation results, with a relative error of less than 8%. This validates the correctness of the micro-motion platform’s compliance model. Theoretical calculated values are slightly lower than simulation values because rigid components in the theoretical model are treated as deformable (despite their high stiffness) in the finite element simulation process.

### 4.2. Kinetostatic Verification

To validate the correctness of the kinetostatic model, a predefined set of trajectories defined by Equation (26) is initially input into the theoretical model using Equation (25) to obtain the corresponding input displacements. Subsequently, these input displacements are applied to the finite element model to obtain the simulated trajectory of the mobile platform. Finally, the simulated trajectory is compared with the predefined trajectory to validate the correctness of the kinetostatic model. In this simulation case, the structural parameters of the micro-motion platform remain consistent with those given in Section 4.1. The position vectors for the transformations between different coordinate systems can be measured in the 3D model, as presented in Table 4.

(26) x=L1sinγcospγy=L1sinγsinpγz=L1cosγL1=1E−4 m, 0≤γ≤π, p=10

**Figure 7:**
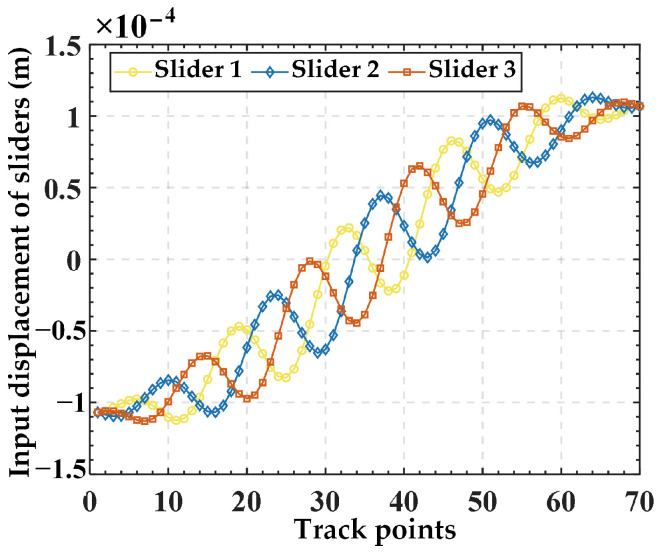
The input displacements for each slider obtained from the spiral trajectory.

**Figure 8:**
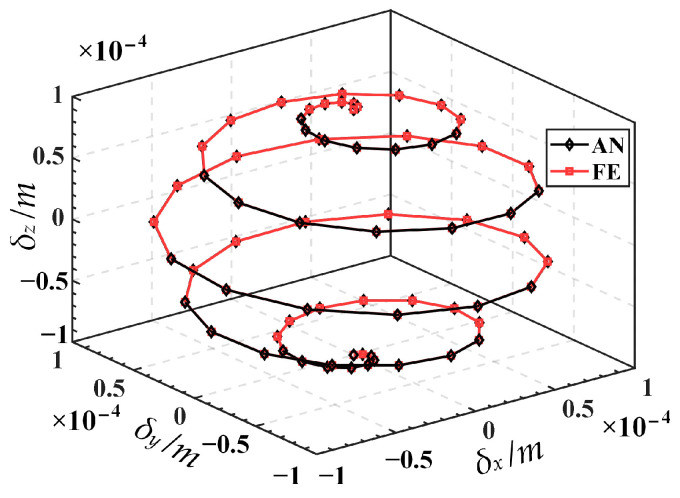
Comparison of the given trajectory (AN) and the simulation trajectory (FE).

**Figure 9:**
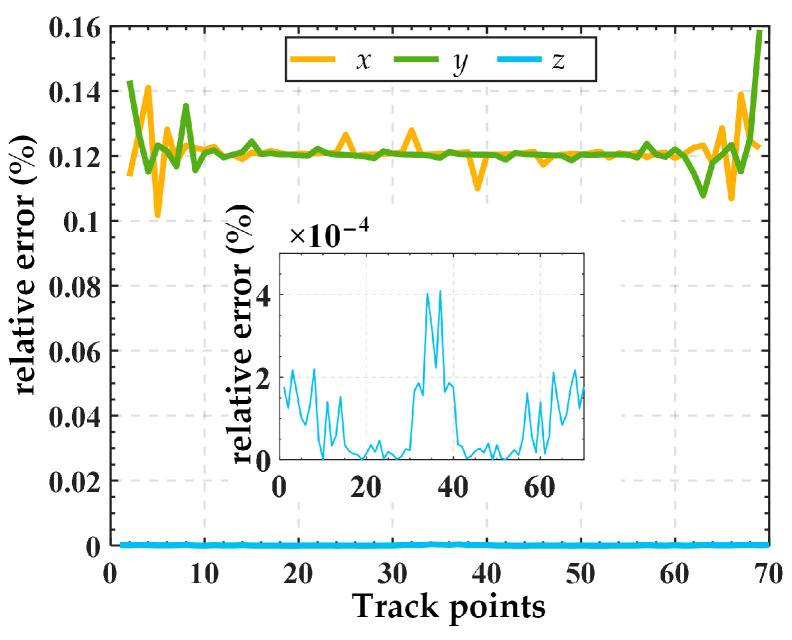
Relative error of displacements between the given trajectory (AN) and the simulation trajectory (FE).

Substituting Equation (26) into Equation (25) yields input displacements, as illustrated in Figure 7. Then, the input displacements are applied to the finite element model, resulting in the simulated output trajectory. Finally, the given trajectory (AN) is compared with the simulation trajectory (FE), as shown in Figure 8. The relative errors in displacement along the *x*, *y*, and *z* directions are illustrated in Figure 9. The results indicate good alignment between the provided trajectory and the simulated trajectory. The relative errors in displacement along the *x* and *y* directions are within 0.16%, while the relative error in the *z* direction is only approximately 0.0004%.

### 4.3. Performance Analysis of Kinetostatic

An important characteristic in the design of the generalized 3-PSS compliant parallel micro-motion platform is the variability in its guide rail inclination angle. This feature expands the range of motion performance for such micro-motion platforms. Hence, this section will further explore the impact of the variation in guide rail inclination angle on the kinetostatic performance of the micro-motion platform. The main focus is on two aspects: (1) the variation in the maximum input displacements required for different guide rail inclination angles under the same output displacement and (2) the variation in the micro-motion platform’s output displacements for different guide rail inclination angles under the same input displacement. The spatial spiral trajectory corresponding to Equation (26) in Section 4.2, along with an additional set of planar circular trajectories corresponding to Equation (27), are taken as examples for separate analyses.

(27) x=L2sinωty=L2sinωtz=L2L2=1E−4 m, ω=π/4

**Figure 10:**
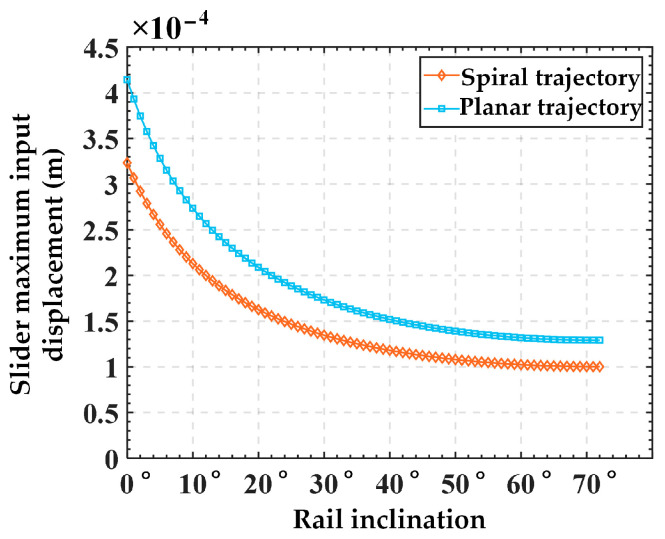
Variation in maximum input displacement for slider with different guide rail inclination angle under the same output displacement.

We substitute the spatial spiral trajectory corresponding to Equation (26) and the planar circular trajectory corresponding to Equation (27) into Equation (25), respectively, and continue to analyze the input displacements obtained under different guide rail inclination angles. The required maximum input displacement variation curves of the sliders for each guide rail inclination angle corresponding to the two trajectories are depicted in Figure 10. It can be seen from Figure 10 that under the condition of achieving the same output displacement, when the guide rail inclination angle *θ* increases from 0° to the angle *α* between the PSS link and the fixed platform (approximately 72°), the required maximum input displacement for the sliders gradually decreases. As is well known, a significant drawback of piezoelectric-driven systems is its limited stroke, and the selection of a large stroke needs to be at the expense of larger structural dimensions and higher costs. Thus, it can be seen that for the same output displacement requirements of the platform, if the guide rail inclination angel is designed to be large, just selecting a piezoelectric stage with a smaller stroke (corresponding to a smaller size and lower cost) can meet the requirements. Therefore, in practice, if the travel of the piezoelectric stage is limited and insufficient to achieve the required maximum output displacement, it is suggested to appropriately increase the inclination angle of the guide rail to meet the requirements.

**Figure 11:**
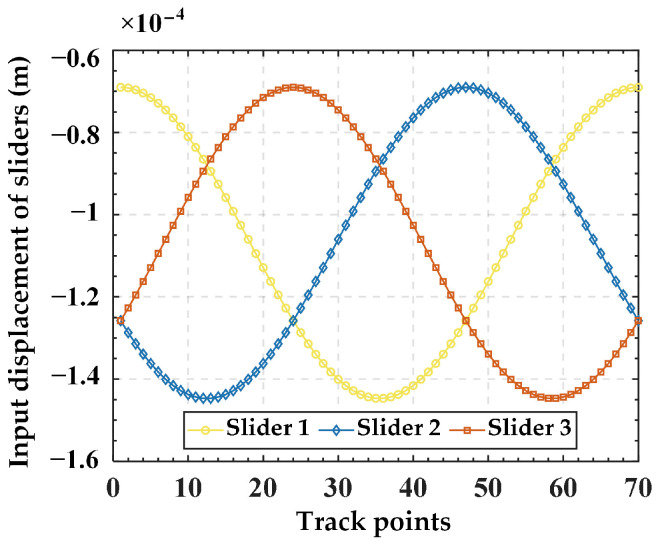
The input displacements for each slider obtained from the planar trajectory.

**Figure 12:**
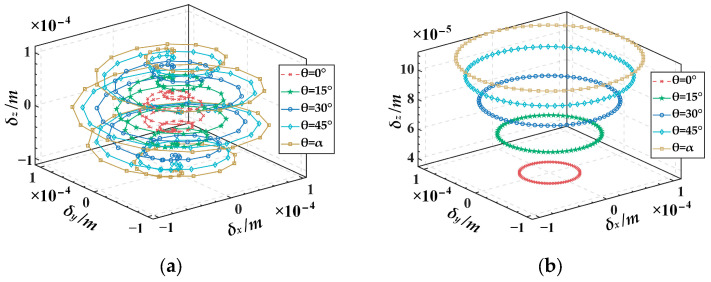
The output displacements of the micro-motion platform for various guide rail inclination angles under the same input displacement: (**a**) spiral trajectory, (**b**) planar trajectory.

Furtherly, we analyze the variation in platform output displacement resulting from different guide rail inclination angles under the same slider input displacement. For simplicity, only five sets of guide rail inclination angles are selected: 0°, 15°, 30°, 45°, and *α* (72°). Here, spatial spiral trajectory and planar circular trajectory are used again for analysis. The input displacements of the slider corresponding to the spiral trajectory are chosen from Figure 7 in Section 4.2, while those corresponding to the planar trajectory are obtained by substituting Equation (27) into Equation (25) (with a guide rail inclination angle of 45°), as illustrated in Figure 11. The output trajectories of the mobile platform for different guide rail inclination angles corresponding to the two input displacements are illustrated in Figure 12a,b. It can be seen from Figure 12 that under the condition of the same input displacement, as the guide rail inclination angle increases gradually from 0° to *α*, the output displacements of the mobile platform in the *x*, *y,* and *z* directions all increase. Therefore, it can be inferred that, under the condition of the same input displacement, increasing the guide rail inclination angle can expand the workspace of the mechanism. However, it is essential to note that the guide rail inclination angle is not necessarily the greater the better. This is because under the same input displacement, increasing the output displacement will lead to a higher output resolution of the mechanism, thereby reducing the output motion precision of the mechanism. Therefore, in practical applications, if the stroke of the piezoelectric stage is predetermined, it is recommended to choose a smaller guide rail inclination angle on the premise of meeting the platform’s output range, to achieve higher precision in the motion of the mechanism. Taking the spatial spiral trajectory in Figure 8 as an example, it can be seen from Figure 10 that if the motion stroke of the piezoelectric stage is 200 μm, a choice of 10° of the guide rail inclination angle may be appropriate.

## 5. Conclusions

This paper introduced a generalized 3-PSS compliant parallel micro-motion platform and investigated its compliance and kinetostatic models. The conclusions are as follows:

1. The compliance model of the micro-motion platform was established using the coordinate transformation method. The compliance model was verified through finite element simulation with an example. The results showed the relative error between theoretical calculation results and the simulation values on the main diagonal elements was less than 8%, indicating the correctness of the compliance model.
2. The governing equation of the equivalent spring system for the micro-motion platform was established based on Hooke’s Law. Then, the kinetostatic model of the generalized 3-PSS compliant parallel micro-motion platform was established using the compliance matrix method. Based on this model, the mapping relationship between the input and output displacements of the micro-motion platform was further derived. Finite element simulation results demonstrated that the relative errors of displacement in the *x* and *y* directions were within 0.16%, while the relative error in the *z* direction was merely about 0.0004%. This strong consistency validated the correctness of the kinetostatic model of the micro-motion platform.
3. The results on the influence of guide rail inclination angel variation on the kinetostatic performance of the micro-motion platform indicate that (1) for the same output trajectory, as the guide rail inclination angel *θ* increases from 0° to *α* (the angle between the PSS link and the fixed platform), the required maximum input displacement for the sliders gradually decreases. It can be inferred that when meeting the same platform output displacement requirements, designing a larger guide rail inclination angle allows for the use of a piezoelectric stage with a smaller stroke range (corresponding to smaller dimensions and lower costs) to fulfill the specifications. (2) Under the same input displacement, as the guide rail inclination angle gradually increases from 0° to *α*, the output displacements of the mobile platform in the *x*, *y*, and *z* directions all increase. Therefore, it can be inferred that, under the same input displacement, increasing the guide rail inclination angle can expand the workspace of the mechanism. However, this comes at the cost of sacrificing some output motion precision. This analysis provides guidance for the rational design of such micro-motion platforms.

## Figures and Tables

**Table 1:** The fundamental parameters of the generalized 3-PSS compliant parallel micro-motion platform.

Parameter	Value (mm)	Parameter	Value (mm)
r	25	l	65
R	45	d	3.5

**Table 2:** The material characteristics and dimensional parameters of the compliant spherical hinge.

Density ρ/(kg/m^3^)	Elastic Modulus E/(Gpa)	Poisson’s Ratio ν	Minimum Thickness (mm)	Cutting Radius (mm)
8000	128	0.3	1	2.5

**Table 3:** Analysis results of compliance of the generalized 3-PSS compliant parallel micro-motion platform.

Compliance	AN	FE	Relative Error (%)
$C_{\theta_{x},m_{x}}$ (rad/(N·m))	1.0912 × 10^−3^	1.1747 × 10^−3^	7.108
$C_{\theta_{y},m_{y}}$ (rad/(N·m))	1.0912 × 10^−3^	1.1747 × 10^−3^	7.108
$C_{\theta_{z},m_{z}}$ (rad/(N·m))	5.4606 × 10^−3^	5.8785 × 10^−3^	7.109
$C_{\delta_{x},f_{x}}$ (m/N)	4.9958 × 10^−6^	5.3785 × 10^−6^	7.115
$C_{\delta_{y},f_{y}}$ (m/N)	4.9958 × 10^−6^	5.3785 × 10^−6^	7.115
$C_{\delta_{z},f_{z}}$ (m/N)	8.0793 × 10^−9^	8.7215 × 10^−9^	7.363

**Table 4:** The measured position vector parameters.

Parameter	Value (mm)	Parameter	Value (mm)
$\Delta d_{1}$	24.23	$\Delta d_{2}$	−8.12
$\Delta l_{1}$	37.55	$\Delta l_{2}$	−69.6

## Data Availability

Data are contained within the article.

## Appendix A

The calculation formula for a right-circular compliant spherical hinge in its own coordinate system is as follows:

$$\left\{\begin{array}{l} C_{\theta_{x},m_{x}}=\frac{64\left(1+\nu \right)}{\pi E}\int_{0}^{2r_{0}}\frac{1}{t{\left(x\right)}^{4}}dx\\ C_{\theta_{y},m_{y}}=\frac{64}{\pi E}\int_{0}^{2r_{0}}\frac{1}{t{\left(x\right)}^{4}}dx,C_{\theta_{y},f_{z}}=-\frac{64r_{0}}{\pi E}\int_{0}^{2r_{0}}\frac{1}{t{\left(x\right)}^{4}}dx\\ C_{\theta_{z},m_{z}}=C_{\theta_{y},m_{y}},C_{\theta_{z},f_{y}}=-C_{\theta_{y},f_{z}}\\ C_{\delta_{x},f_{x}}=\frac{4}{\pi E}\int_{0}^{2r_{0}}\frac{1}{t{\left(x\right)}^{2}}dx\\ C_{\delta_{y},m_{z}}=C_{\theta_{z},f_{y}},C_{\delta_{y},f_{y}}=\frac{64}{\pi E}\int_{0}^{2r_{0}}\frac{x^{2}}{t{\left(x\right)}^{4}}dx+\frac{k\left(1+\nu \right)}{\pi E}\int_{0}^{2r_{0}}\frac{1}{t{\left(x\right)}^{2}}dx\\ C_{\delta_{z},m_{y}}=C_{\theta_{y},f_{z}},C_{\delta_{z},f_{z}}=C_{\delta_{y},f_{y}} \end{array}\right.$$

where E represents the elastic modulus of the material, ν is the Poisson’s ratio of the material, k is the shear shape factor (taking *k* = 10/9), $r_{0}$ is the cutting radius of the straight cylindrical compliant spherical hinge. $t\left(x\right)$ is the thickness function describing the compliance of the spherical hinge along the *x*-axis, and its expression is as follows:

$$t\left(x\right)=t_{\min}+2(r_{0}-\sqrt{x\left(2r_{0}-x\right)})(x\in \left[0,2r_{0}\right])$$
